# micRoclean: an R package for decontaminating low-biomass 16S-rRNA microbiome data

**DOI:** 10.3389/fbinf.2025.1556361

**Published:** 2025-05-08

**Authors:** Rachel Griffard-Smith, Emily Schueddig, Diane E. Mahoney, Prabhakar Chalise, Devin C. Koestler, Dong Pei

**Affiliations:** ^1^ Department of Biostatistics & Data Science, University of Kansas Medical Center, Kansas City, KS, United States; ^2^ School of Nursing, University of Kansas Medical Center, Kansas City, KS, United States

**Keywords:** microbiome, 16S-rRNA, decontamination, metabolomics, low-biomass, cross-contamination

## Abstract

In 16S-rRNA microbiome studies, cross-contamination and environmental contamination can obscure true biological signal. This contamination is particularly problematic in low-biomass studies, which are characterized by samples with a small amount of microbial DNA. Although multiple methods and packages for decontaminating microbiome data exist, there is no consensus on the most appropriate tool for decontamination based on the individual research study design and how to quantify the impact of removing identified contaminants to avoid over-filtering. To address these gaps, we introduce micRoclean, an open-source R package that contains two distinct microbiome decontamination pipelines with guidance on which to select based on the downstream goals of the research study and study design. This package integrates and expands on existing packages for microbiome decontamination and analysis for convenience of users. Furthermore, micRoclean also implements a filtering loss statistic to quantify the impact of decontamination on the overall covariance structure of the data. In this paper, we demonstrate the utility of micRoclean through implementation on example data, illustrating that micRoclean effectively and intuitively decontaminates microbiome data. Further, we demonstrate through a multi-batch simulated microbiome sample that micRoclean matches or outperforms tools with similar objectives. This package is freely available from GitHub repository rachelgriffard/micRoclean.

## 1 Introduction

Microbiome profiling through 16S-rRNA sequencing is used commonly to study the bidirectional relationship between the microbiome and disease status, diet, exercise, pollution, etc. (Sun, 2023; Riquelme et al., 2019; Luan et al., 2024). These studies often use high-biomass samples such as stool and saliva; however, in recent years, interest has increased in profiling low-biomass samples such as blood, plasma, and skin (Urbaniak et al., 2016; Wang et al., 2022; Mrázek et al., 2019). In these low-biomass microbiome studies, contaminant bacteria can often obscure true biological signal to a greater degree when compared to high-biomass studies. This problem arises due to the inherent lower amount of microbial DNA initially present in low-biomass samples. Consequently, in these low-biomass samples, a well-noted source of technical variability in microbiome data, contaminant bacteria, often represent a greater proportion of the overall signal (Salter et al., 2014). These contaminant bacteria arise from two main sources: (1) cross-contamination between samples and (2) contaminant DNA from the surrounding environment (Eisenhofer et al., 2019). To improve the likelihood of identifying true biological signal rather than this contaminant DNA, it is necessary to remove suspected contamination prior to the analysis of microbiome data. This removal step is particularly important for low-biomass samples, as they are inherently at a higher risk of contamination.

Current methods to decontaminate microbiome data can broadly be classified into three main categories: blocklist, sample-based, and control-based (Hülpüsch et al., 2023). Blocklist methods detect and entirely remove the feature(s) contained in lists that are previously identified in the literature as common contaminants. Sample-based methods identify contaminant features based on their relative abundance, removing features that are different between batches. Control-based methods identify contaminant features based on abundance in negative control samples. Using one or multiple of these methods, current software packages aim to decontaminate microbiome samples. For example, GRIMER (Piro and Renard, 2023) implements the MGnify tool from EMBL-EBI (Richardson et al., 2022) to identify blocklist contaminants based on their known source in previous studies within a Graphic User Interface dashboard. Most existing tools such as these remove entire features that are identified as contaminants, such as the well-established decontam package (Davis et al., 2018). The decontam package combines control- and sample-based contaminant identification and removes entire features tagged as contaminants. Meanwhile, other methods aim to remove only the proportion of features identified as contamination. This partial removal is implemented in packages such as MicrobIEM (Hülpüsch et al., 2023), microDecon (McKnight et al., 2019), and SCRuB (Austin et al., 2023). MicrobIEM, microDecon, and SCRuB packages all leverage a control-based decontamination method.

**FIGURE 1:**
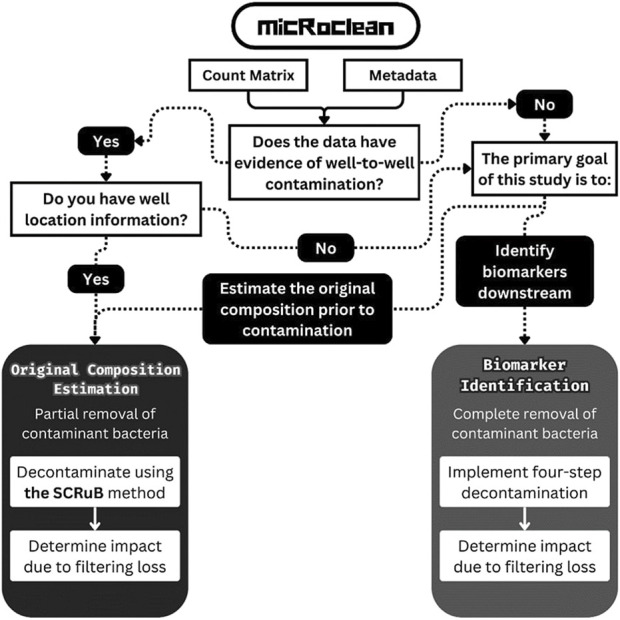
Flowchart for implementation of *micRoclean* package.

While methods for identifying and removing contamination within microbiome data are plentiful, there is no consensus on: (i) situationally, which methods and tools are most appropriate to use and (ii) how to quantify the removal to avoid overfiltering. To address these gaps, we introduce micRoclean, an open-source R package housing one function with two pipelines (Figure 1) aimed at decontaminating low-biomass microbiome data. The Original Composition Estimation pipeline aims to most closely estimate the original microbiome composition prior to contamination, while the Biomarker Identification pipeline aims to strictly remove all likely contaminant features to minimize the likelihood downstream biomarker identification analyses are impacted by these contaminant features. Within micRoclean, users have flexibility and guidance to choose the most appropriate decontamination pipeline based on their primary research goal. Furthermore, micRoclean provides a measurement of decontamination that quantifies the impact of feature removal and provides insight into potential over-filtering through a filtering loss statistic. In what follows, we describe the architecture of the micRoclean package and demonstrate the utility of this tool by decontaminating a multi-batch simulated microbiome dataset and a real-world blood plasma microbiome dataset.

## 2 Methods

The micRoclean package and function contain two distinct pipelines for decontaminating 16S-rRNA sequencing samples.

### 2.1 Input data

The data required as input for the micRoclean package are a sample ( n ) by features ( p ) count matrix generated from 16S-rRNA sequencing and a metadata matrix with samples ( n ) rows generated from 16S-rRNA sequencing data. This metadata defines the samples in the count matrix and contains columns specifying if the sample is a control and the group name. Optionally, the user can include batch and sample well location columns within the metadata.

### 2.2 Well-to-well contamination

Well-to-well leakage is a common form of contamination where biological samples leak into controls. For batches where users do not have well location information, the well2well function is implemented automatically within the micRoclean function and assigns pseudo-locations in a 96-well plate. This is accomplished by assuming a common order of samples vertically or horizontally. This function then estimates the proportion of each control that originates from a biological sample to estimate well-to-well leakage by leveraging the SCRuB package spatial functionality within the SCRuB function (Austin et al., 2023). The micRoclean package extracts and reshapes this from the source code of the SCRuB functionality, which does not include this as output for the general user. If the level of well-to-well contamination is higher than 0.10, the function will return a warning message indicating to the user that they should obtain the well location information for their data and run through the Original Composition Estimation pipeline, as this pipeline can account for well-to-well leakage contamination.

### 2.3 Filtering loss (FL)

The output from both pipelines includes a filtering loss (FL) value. This statistic was first introduced by Smirnova et al. (2019) as a metric for their permutation-based filtering method for removal of full features. However, as the FL value quantifies the contribution the filtered features to the overall covariance, we use this statistic to quantify the impact of suspected contaminant feature removal on the overall covariance structure of the samples. Furthermore, we use this statistic in a novel way to quantify the impact on covariance not only from full feature removal as within the original use case but partial removal of reads—such as implemented in the Original Composition Estimation pipeline.

For a count matrix $X,{\left|\left|X^{T}X\right|\right|}_{F}^{2}=\Sigma_{j=1}^{p}{\left(x_{j}^{T}x_{j}\right)}^{2}+\Sigma_{i>j}{\left(x_{i}^{T}x_{j}\right)}^{2}$ approximates the total covariance. We can compare the covariance before and after filtering a count matrix to consider the impact of filtering. FL is defined in Equation 1 as such:

$$FL\left(J\right)=1-\frac{{\left|\left|Y^{T}Y\right|\right|}_{F}^{2}}{{\left|\left|X^{T}X\right|\right|}_{F}^{2}}$$ (1)

where X is the n x p pre-filtering, full count matrix and Y is the n x q post-filtering count matrix resulting from the partial removal of reads or whole removal of features after applying the decontamination method. As full features may or may not be removed, q≤p .

$FL\left(J\right)$ is therefore a ratio of the filtered to full covariance matrices. As Y is a subset of the full count matrix X , $FL\left(J\right)$ represents the contribution of the removed partial or full features to the overall covariance. Values closer to 0 indicate low contribution to the overall covariance of X , while values closer to 1 indicate high contribution and could be a warning sign of over-filtering. Further discussion and example of the FL statistic is within Supplementary Appendix SA13.1.

### 2.4 Original Composition Estimation pipeline

The first pipeline, research_goal = “orig.composition,” is ideal for aiming to characterize samples’ original compositions as closely as possible to the sample composition prior to contamination. The decontamination step in Original Composition Estimation pipeline implements the SCRuB method (Austin et al., 2023). The results from this pipeline include the filtered count matrix from the SCRuB method and the filtering loss value.

The Original Composition Estimation pipeline expands SCRuB by directly decontaminating multiple batches within one line of code. Using SCRuB directly with multiple batches of data, users must separate the count matrices by batch, run through SCRuB, and recombine the resulting count matrices. In this process, users may mistakenly run multiple batches together due to the lack of functionality support within SCRuB for multiple batches. This misuse of the method results in incorrect decontamination. Within the micRoclean Original Composition Estimation pipeline, the tool automatically runs SCRuB for multiple batches within the same one line of code by implementing information from the included metadata: the micRoclean function splits the data to decontaminate by batch, appends the count matrices back together, returning the full, properly decontaminated count matrix.

The Original Composition Estimation pipeline is the most appropriate choice if the user is concerned about well-to-well contamination and has well location information as SCRuB decontamination can account for leakage between samples (Austin et al., 2023). This pipeline is also most appropriate if there is only one batch of samples, in contrast to the Biomarker Identification pipeline which requires multiple batches to decontaminate.

### 2.5 Biomarker Identification pipeline

Initiated by setting research_goal = “biomarker,” the Biomarker Identification pipeline derives its underlying architecture from the four-step pipeline first introduced by Zozaya-Valdés et al. to decontaminate low-biomass cell-free microbial DNA (Zozaya-Valdes et al., 2021). The methods within this architecture have been adjusted to be more appropriate for compositional data from 16S-rRNA sequencing. This function is ideal for (i) users whose primary research goal downstream of decontamination is to identify biomarkers, and therefore want stringent removal, and/or (ii) users who do not have DNA-negative control samples within each of their batches, which is required for running the Original Composition Estimation pipeline and other control-based decontamination methods. This pipeline implements multiple methods to identify contamination through four separate steps. Through this stringent removal, users can be more confident that the unfiltered features are not contamination. Therefore, the decontaminated data matrix produced by our Biomarker pipeline enables downstream statistical testing to more confidently identify potential biomarkers, as the observed differences reflects better true biological conditions rather than contamination.

The first step is a sample-based decontamination method where features with different abundance across different batches are tagged as contaminant features. These are tagged based on significantly different compositions across batches using the Analysis of Compositions of Microbiomes with Bias Correction (ANCOM-BC) method (Lin and Peddada, 2020). The second step is a control-based method where features higher in negative controls and lower in samples are tagged as contaminant features. To accomplish this, the decontam prevalence method is used (Davis et al., 2018). In the third step, features that have different abundance across different batches for technical replicates are tagged as contaminant features. This is a sample-based decontamination method which uses Cohen’s unweighted kappa statistic to compare the agreement between technical replicates across batches and remove those with disagreement (Gamer et al., 2019). In the fourth and final step, features are tagged as contaminants if they appear on user-defined blocklists of previously known contaminant features. If users do not have their own blocklists, it is suggested to use the publicly available blocklist published from Eisenhofer et al. and available for ease of use within the micRoclean package data (Eisenhofer et al., 2019). The provided blocklist is at the genus level, but users have flexibility to include multiple levels of taxonomic rank if they choose to define their own blocklists.

The resulting filtered count matrix removes entire features that are identified as contaminants based on a user-defined number of steps in which a feature is identified as a contaminant. The results include this filtered count matrix, the list of removed features, a matrix identifying which steps identified each feature as a contaminant, and the associated filtering loss value.

### 2.6 Benchmarking micRoclean

#### 2.6.1 Multi-batch simulated microbiome dataset

The multi-batch simulated dataset used for benchmarking is created by simulating two batches of 16S-rRNA sequencing samples with known contamination. Batch 1 contains five samples and two DNA-extraction negative controls; batch 2 contains five samples—two of which are technical replicates from batch 1—and two DNA-extraction negative controls. The non-extraction control samples were further assigned as “Disease” or “Healthy control” samples, with members of each group represented within the two batches. Specifically, Batch 1 contains two ‘Healthy control’ samples—S1A, S1B—and three “Disease” samples—S3, S4, S5. Batch 2 contains four “Healthy control” samples—S2A, S2B, S7, S8—and one “Disease” sample—S6.

The simulated data were created with ten genera which are commonly identified in the human microbiome simulated as the true biological signal—*Bacteroides, Bifidobacterium, Roseburia, Butyrivibrio, Parabacteroides, Peptostreptococcus, Alistipes, Eubacterium, Faecalibacterium* and *Ruminococcus*. These simulated features were intentionally selected to align with real genera names to enable direct matching against existing blocklists utilized by micRoclean and GRIMER methods. Furthermore, the first five of these non-contaminant genera were simulated to have differential abundance between the “Disease” and “Healthy control” simulated samples, while the other five were not. These data contain 10 samples, 4 DNA-negative controls, and 110 ASVs in total.

Technical replicate counts were generated by sampling from a uniform distribution between 10 and 100 [U(10,100)]. Independent Gaussian noise, reflecting measurement variability, was subsequently added to each count from a normal distribution centered at zero with a standard deviation of 10 [N(0,10)]. Similarly, non-replicates from the first batch were drawn from a uniform distribution $U\left(20,90\right)$ with independent Gaussian noise $N\left(0,10\right)$ ; and non-replicates from the second batch were drawn from a uniform distribution $U\left(30,100\right)$ with independent Gaussian noise $N\left(0,10\right)$ . Contaminant features for the first batch were drawn from a uniform distribution $U\left(0,20\right)$ with independent Gaussian noise $N\left(0,2\right)$ . The second batch’s contaminant features were generated using the same method but with a different random seed.

Contaminant features for the first batch were drawn from uniform distribution $U\left(0,20\right)$ with independent Gaussian noise $N\left(0,2\right)$ . The second batch’s contaminant features were generated using the same method but with a different random seed.

Non-contaminant counts of the features representing an equal proportion were simulated as follows: technical replicates were drawn from a uniform distribution $U\left(10,100\right)$ with independent Gaussian noise from a normal distribution $N\left(0,10\right)$ ; non-replicates from the first batch drawn from a uniform distribution $U\left(20,90\right)$ with independent Gaussian noise $N\left(0,10\right)$ ; and non-replicates from the second batch were drawn from $U\left(30,100\right)$ with independent Gaussian noise $N\left(0,10\right)$ .

Five non-contaminant genera—*Roseburia, Bacteroides, Bifidobacterium, Butyrivibrio,* and *Parabacteroides*—were subjected to group-specific scaling to model differential abundance of potential biomarker features. The “Disease” group counts were multiplied by values sampled from a uniform distribution $U\left(1.5,2.5\right)$ , the “Healthy control” group were multiplied by values from a different uniform distribution $U\left(0.5,0.8\right)$ .

To include contaminants that were dependent between samples and controls within the same batch, a systematic approach was applied with random noise. Using separate random seeds for each batch, this was accomplished by creating a random contamination vector generated from the negative binomial distribution NB(1,0.5). This was added to the associated samples contaminants with a scaling factor from a uniform distribution U(1,1.5) and added to associated negative controls with a larger scaling factor from another uniform distribution U(2,3).

**FIGURE 2:**
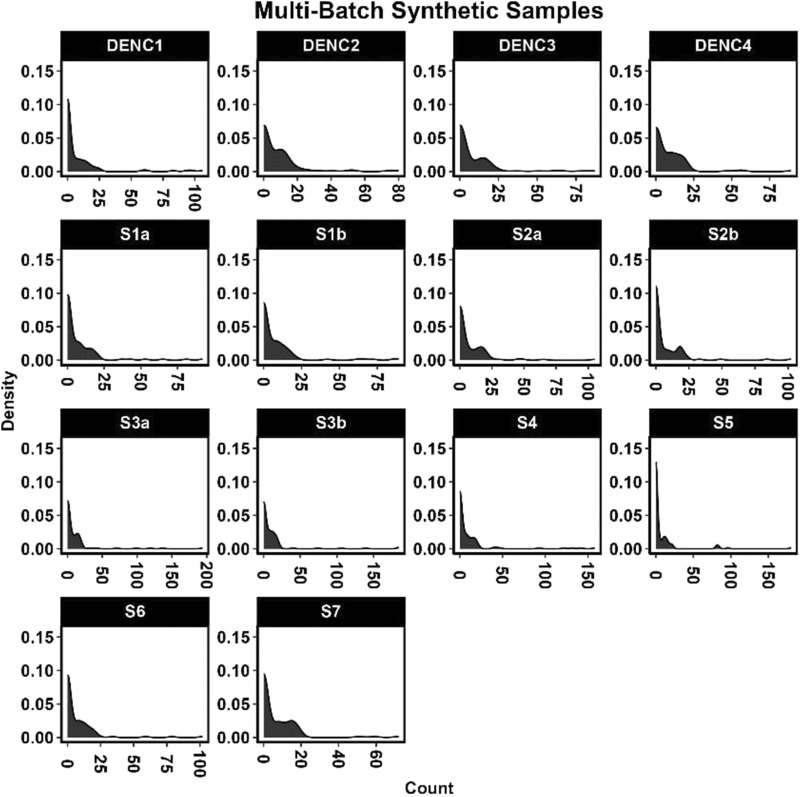
Count distribution of features in multi-batch simulated data.

To model the zero-inflation characteristic of true 16S-rRNA microbiome data within our multi-batch simulated dataset, we implement a probabilistic zeroing mechanism. The probability of a given count being zeroed was calculated in Equation 2 as such:

$$p_{n,p}=1-\frac{x_{n,p}}{\max_{j}\left(x_{n,j}\right)}$$ (2)

resulting in smaller counts within each sample having a higher probability of being set to zero. This was relaxed by multiplying the zero-probability matrix by 0.6. These probabilities are used within a binomial distribution to determine if a feature is set to zero (1) or not (0). The resulting distributions for each simulated sample and DNA-negative control are shown in Figure 2.

**FIGURE 3:**
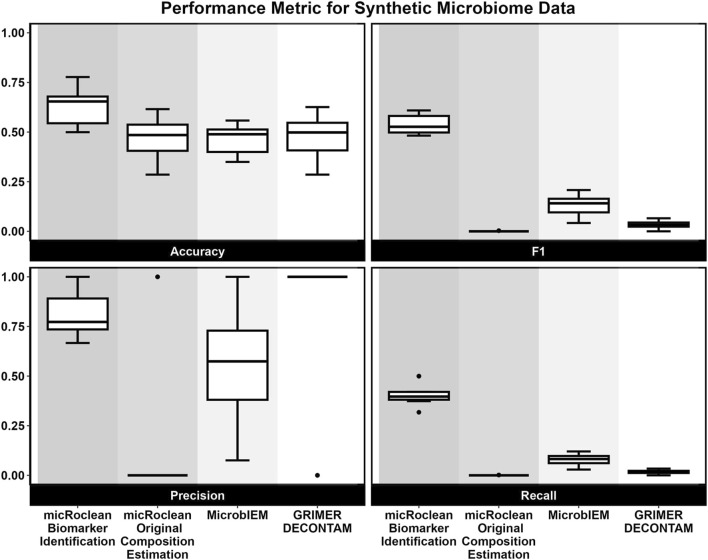
Performance metrics across methods for simulated microbiome data.

For both micRoclean pipelines and the following tools, performance metrics were calculated for decontamination on this synthetic dataset, with a positive case indicating a contaminant. Accuracy, F1, precision, and recall were calculated for all ten samples across all four methods. These results are visualized within Figure 3 and contained within Table 1.

**TABLE 1:** Performance metrics for micRoclean, GRIMER, and MicrobIEM by sample for multi-batch simulated microbiome dataset.

Metric	Method	Batch 1					Batch 2					Average
		1A	2A	3A	4	5	1B	2B	3B	6	7	
Accuracy	micRoclean Biomarker Identification	0.6485	0.5321	0.6801	0.7047	0.6755	0.5812	0.4994	0.7772	0.66	0.5303	0.629
	micRoclean Original Composition Estimation	0.49	0.3708	0.5319	0.5393	0.5647	0.4744	0.3826	0.6155	0.48	0.2855	0.473
	MicrobIEM	0.5037	0.3849	0.4167	0.558	0.5161	0.518	0.394	0.4896	0.4882	0.3497	0.462
	GRIMER	0.4984	0.3821	0.5363	0.5504	0.5704	0.4831	0.3826	0.6259	0.4976	0.2855	0.481
Precision	micRoclean Biomarker Identification	0.7938	0.7317	1	0.7807	0.7453	0.6775	0.6667	1	0.7647	0.9234	0.808
	micRoclean Original Composition Estimation	NaN	NaN	1	NaN	1	NaN	NaN	NaN	NaN	NaN	NA
	MicrobIEM	0.7826	0.5676	0.1638	0.6262	0.3209	0.7632	0.5806	0.0754	0.5593	1	0.544
	GRIMER	1	1	1	1	1	1	NaN	1	1	NaN	1
Recall	micRoclean Biomarker Identification	0.4198	0.4048	0.3177	0.4992	0.3885	0.388	0.3785	0.4205	0.5	0.3736	0.409
	micRoclean Original Composition Estimation	0	0	0.0016	0	0.0016	0	0	0	0	0	0
	MicrobIEM	0.037	0.0945	0.0595	0.1011	0.0984	0.1203	0.0668	0.0291	0.0747	0.0897	0.077
	GRIMER	0.0165	0.018	0.011	0.0241	0.0148	0.0166	0	0.0271	0.0339	0	0.016
F1	micRoclean Biomarker Identification	0.5491	0.5212	0.4822	0.609	0.5108	0.4934	0.4828	0.5921	0.6047	0.5319	0.538
	micRoclean Original Composition Estimation	0	0	0.0031	0	0.0033	0	0	0	0	0	0.001
	MicrobIEM	0.0707	0.162	0.0873	0.174	0.1506	0.2079	0.1198	0.042	0.1317	0.1647	0.131
	GRIMER	0.0324	0.0353	0.0217	0.0471	0.0291	0.0327	0	0.0528	0.0656	0	0.032

#### 2.6.2 Real-world blood plasma microbiome dataset

Real low-biomass cell-free microbial DNA from plasma 16S-rRNA sequencing samples were used from Zozaya-Valdes et al. (2021). These data contain two batches of 126 samples, 36 of which are DNA extraction negative controls initially containing only nuclease-free water. Full methods for data preparation can be found in the original manuscript. Briefly, samples were extracted using the QIAGEN QIAmp Circulating Nucleic Acid Kit. These samples were analyzed in paired-end sequencing on the Illumina MiSeq instrument and then processed using QIIME2 (version 2018.11.0) (Bolyen et al., 2019). The Amplicon Sequencing Variant (ASV) count table was generated using the Silva database (119 SSU Ref NR 99 515F/806R release) (Quast et al., 2013). Metadata information such as DNA extraction batch were recorded for bioinformatic decontamination. The processed count matrix contains 1,792 ASVs. This study used the processed count matrix from the original study (Zozaya-Valdes et al., 2021).

#### 2.6.3 GRIMER

GRIMER is a tool created to facilitate contamination detection in low-biomass microbiome data through an interactive dashboard (Piro and Renard, 2023). This tool requires users to input a count matrix with the option to include metadata and taxonomic information. GRIMER integrates these data and returns an interactive dashboard that provides multiple visual tools to manually identify contamination. Users can include information about feature origin from previous publications from MGnify tool from EMBL-EBI (Richardson et al., 2022) (e.g., sample, environment, etc.). Optionally, users can also choose to implement contamination prediction through the decontam method (Davis et al., 2018). In our benchmark study, this tool is accessed through installation in a conda environment and run via command line interface.

For the multi-batch simulated microbiome data, GRIMER was initiated by inputting the count matrix, taxonomic information, and metadata. The MGnify tool from EMBL-EBI (Richardson et al., 2022) and decontam frequency method with threshold set to 0.1 (Davis et al., 2018) were implemented.

#### 2.6.4 MicrobIEM

MicrobIEM is a control-based decontamination method run through a web-based interactive dashboard that focuses on low-biomass microbiome data (Hülpüsch et al., 2023). A control-based method, the tool considers the ratio of sequences in negative controls versus samples as well as the proportion of negative controls which contain that sequence. Users must manually set the values for these two parameters when using this tool.

For the multi-batch simulated microbiome dataset, the ratio hyperparameter was set to 2 for the batches of negative controls and the span threshold was set to 1. The package default filtering values for minimum reads were used.

## 3 Results

### 3.1 Usage examples

The following usage examples are contained within the micRoclean package vignette to demonstrate basic micRoclean functionality.

To illustrate the usage within the micRoclean usage vignette for the Original Composition Estimation pipeline, we first import one-batch even mock microbial community dilution dataset from Hülpüsch et al. (2023). Because samples were profiled in one batch, the batch contains at least one control, and the primary goal is to characterize the sample prior to contamination, the most appropriate tool for this analysis is the Original Composition Estimation pipeline. After running the micRoclean function with research_goal = “orig.composition,” we return a filtered counts matrix with partial removal of contaminants identified by the SCRuB method (Austin et al., 2023). The corresponding filtering loss value is 0.22, indicating that the counts removed account for around 22% of the overall covariance.

Similarly, to illustrate the usage within the micRoclean usage vignette for the Biomarker Identification pipeline, we first import the real-world blood plasma dataset from Zozaya-Valdes et al. (2021). After running the Biomarker Identification pipeline in the micRoclean function, filtered count matrix is returned with full removal of features that were tagged as contaminants in the user-defined threshold number of the four filtering steps, defaulting to one. Using this default, we find that this removes 1,118 features of the original 1,795 features. The filtering loss statistic associated with this is 0.88, indicating that the removed features accounted for 88% of the overall covariance. Once the user has the Biomarker Identification pipeline method results, these results can be plugged into the visualize_pipeline function to return a Venn diagram identifying the overlapping identification of features as contaminants by step.

### 3.2 Benchmarking micRoclean with multi-batch simulated dataset

Within the multi-batch simulated microbiome dataset, all simulated samples display zero-inflated as observed with microbiome data (Figure 2).

After decontamination, the Biomarker Identification pipeline retained 62 features, removing 48 whole features tagged as contaminants in at least one of the filtering steps. The filtering loss statistic for this removal is 0.133. Of the ten non-contaminant features, the Biomarker Identification pipeline retained nine.

After decontamination with the Original Composition Estimation pipeline, this method removed 2 counts and retained all features. The filtering loss statistic is $3.25\times 10^{-6}$ . Of the ten non-contaminant features, the Original Composition Estimation pipeline retained ten.

After decontamination, the MicrobIEM method removed 1,111 reads from 7 samples and 13 features, fully removing 10 features. The filtering loss statistic for is 0.161 and indicates that the removed features account for 16% of the covariance in the full count matrix. Of the ten non-contaminant features, MicrobIEM retained nine.

For decontamination, GRIMER returns a Graphic User Interface report to explore contamination within this dataset. The dashboard did identify that the ten non-contaminant genera as most likely host-associated and that the majority of other contaminant simulated genera were likely contaminants from the MGnify tool from EMBL-EBI (Richardson et al., 2022), demonstrating its usefulness for individual feature analysis. The decontam (Davis et al., 2018) results provide a prediction on whether the feature is a contaminant. Used in conjunction with the MGnify results, this information can be implemented in a line by line, manual decontamination. To compare this tool, we exported the predictions of the decontam frequency method and considered the filtered count matrix as those which were not tagged as contaminants at the genus-level. Using this method, two features were fully removed with a filtering loss of 0.001. Of the ten non-contaminant features, the GRIMER decontam method retained all ten.

#### 3.2.1 Method comparison

Performance metrics were calculated with a positive case indicating a contaminant. Accuracy, F1, precision, and recall were calculated for all ten samples across all four methods, and these results are contained within Table 1 and visualized in Figure 3.

Our Biomarker Identification pipeline had the highest accuracy, F1, and recall on average across the methods. Compared to existing tools, our Biomarker Identification method demonstrated higher recall, indicating that it more effectively captures a greater proportion of true contaminants (fewer false negatives). These results are visualized in Figure 3.

The Original Composition Estimation pipeline removed less contaminant reads than other methods but maintained a comparable accuracy to the MicrobIEM and GRIMER decontam methods.

### 3.3 micRoclean performance on real world blood-plasma dataset

**FIGURE 4:**
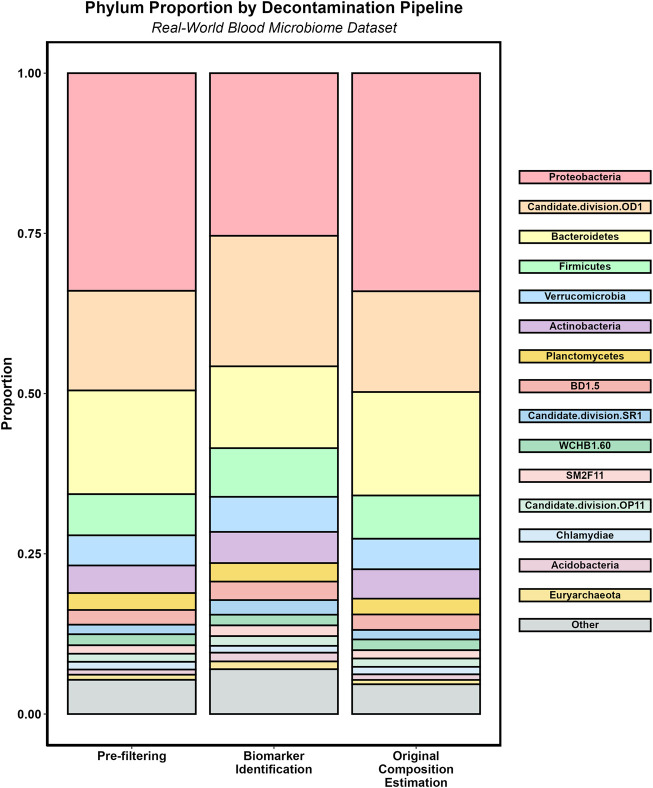
Sample Phylum proportions by decontamination pipeline.

As an example of the usage of micRoclean using a real data, we analysed a blood-plasma 16S-rRNA sequencing dataset originating from Zozaya-Valdes et al. using both the Original Composition Estimation pipeline and the Biomarker Identification pipeline (Zozaya-Valdes et al., 2021). A comparison figure of the composition at the Phylum level pre-filtering and post-filtering with both pipelines is visualized in Figure 4.

When using the Biomarker Identification pipeline with the threshold set at default of 1, the resulting filtered count matrix retains 667 features, fully removing 1,118 features as contaminants. Step 1 identified two features as contaminants, step 2 identified 900 features, step 3 identified 1 feature, and step 4 identified 367 features based on the default blocklist. The resulting filtering loss for this method is 0.88, indicating that one or more of the features removed contributed highly to the covariance of the pre-filtered count matrix.

When using the Original Composition Estimation pipeline to analyze these data, the resulting filtered count matrix retains 1,665 partial or full features, fully removing 130 features as contaminants. The filtering loss from this method is 0.01.

## 4 Discussion

As low-biomass microbiome samples are more commonly analyzed and contamination needs to be accounted for, micRoclean provides clarity and direction on (i) what filtering method is most appropriate based on the research design and goals and (ii) implement a filtering loss statistic to provide insight into the impact of filtering.

### 4.1 Benchmarking micRoclean with multi-batch simulated dataset

In binary classification tasks for contaminant removal such as with this multi-batch simulated dataset, the micRoclean Biomarker Identification pipeline demonstrated superior performance compared to the Original Composition Estimation pipeline, MicrobIEM, and GRIMER decontam. Specifically, micRoclean achieved higher accuracy—correctly classifying features as contaminants or not—; F-1 score—balancing contaminant identification and biological signal in unbalanced, small biological signal-cases—; and recall—ensuring that contaminant features are more likely to be identified.

Unless only implementing the decontam method, and while the GRIMER tool can be useful for initial data exploration or for exploration by a domain expert, due to (i) the inherent bias of the personnel completing the manual decontamination, (ii) the bias towards studied features in MGnify, and (iii) the time intensive nature of this process of going through each feature at the genera level by hand, GRIMER tool lacks the replicability and convenience of a tool such as micRoclean.

MicrobIEM, while providing an interactive tool with a user-friendly interface, does not outperform either the Original Composition Estimation or Biomarker Identification pipeline in decontamination tasks. Furthermore, the online tool can only handle smaller datasets with the user-friendly interface.

The Original Composition Estimation pipeline is most appropriate for data that has mixing of contaminant and non-contaminant counts in the same features, as opposed to this simulation data that has features that are binary: contaminant or non-contaminant.

### 4.2 micRoclean performance on real-world dataset

The resulting post-filtering count matrix on the same real-world low-biomass blood plasma microbiome dataset from the Biomarker Identification pipeline and the Original Composition pipeline greatly differed. The filtering loss statistic indicates that the Original Composition pipeline retained nearly all the features contributing to the covariance of the initial pre-filtering dataset, whereas the Biomarker Identification pipeline removed full features that accounted for 88% of the variance. As the Biomarker Identification pipeline is designed to have more stringent removal, this difference makes sense.

### 4.3 Limitations and future directions

For datasets with large batch effect where a feature is high in all samples of one batch and low in all samples of another batch or vice versa, then this feature would be incorrectly removed using the Biomarker Identification pipeline. Similarly, if this pattern of opposite abundance across batches within the technical replicates across batches—not due to contamination—then the feature would be incorrectly removed. Our Biomarker Identification pipeline is inherently going to cause more stringent removal and full feature removal, likely including some features that may be partially true biological signal and partially contamination. This is a limitation for this pipeline. If this potential removal is a concern, the Original Composition Estimation pipeline should be used and can parse out proportions of features expected to be contamination using partial removal.

Although existing decontamination strategies for 16S microbiome data that use traditional statistical framework exist, advanced machine learning approaches are increasingly implemented across diverse biomedical domains (Camacho et al., 2018; Namkung, 2020; Jin et al., 2022; Li et al., 2022; Wang et al., 2022; Zhao et al., 2022; Hyeonseo Hwang et al., 2024; Przymus et al., 2024; Yue et al., 2024; Zhao et al., 2024; Wang et al., 2025; Zhao et al., 2025).

For future work, machine learning models as Support Vector Machines or Neural Networks show promise for identifying likely contamination in microbiome data. Both machine learning models have shown promise in identifying and characterizing the underlying structure of patterns in high-throughput sequencing biological data (Yang, 2004; Barbero-Aparicio et al., 2023). Incorporating these advanced machine learning techniques into 16S rRNA-sequencing decontamination pipelines offers promising avenues for future work.

### 4.4 Conclusion

The micRoclean package provides users with two pipelines for decontaminating 16S-rRNA sequencing data. Furthermore, it implements a filtering loss statistic originally designed for the complete removal of species to quantify both full and partial removal filtering. Finally, through analyses on a simulated microbiome dataset with known contamination, we have demonstrated that micRoclean performs as well or outperforms similarly positioned tools for decontaminating low-biomass microbiome data.

## 5 Software availability

The micRoclean package is available via GitHub in the repository https://github.com/rachelgriffard/micRoclean. Information about installation and usage can be found in the README file and the vignette contained within this repository. Sample data can be found in the vignette folder. Users can run this through the vignette R Markdown tutorial using the data contained within the vignette folder.

## Data Availability

The original contributions presented in the study are included in the article/Supplementary Material, further inquiries can be directed to the corresponding authors.
