# Supplementary material for: micRoclean: an R package for decontaminating low-biomass 16S-rRNA microbiome data
Source: Front Bioinform. 2025 May 8;5:1556361. doi: 10.3389/fbinf.2025.1556361 (PMC12095030; doi:10.3389/fbinf.2025.1556361)
Supplement: Supplementary file 1 [file DataSheet1.docx]

1. **Appendix**
   1. **Filtering Loss (FL)**

The filtering loss (FL) statistic considers the difference between the covariance matrices of the pre-filtering count matrix $X$ and the post-filtering count matrix $Y$. This comparison is accomplished through considering the ratio of the squared Frobenius norms of the pre- and post-filtering count matrices.

For a pre-filtering $n x p$ count matrix $X, \left| \left| X^{T}X \right| \right|_{F}^{2}$approximates the total covariance through the square of the Frobenius norm of pre-filtering count matrix $X$. Similarly, for post-filtering $n x q$ count matrix $Y$—which is a subset of $X$—the covariance is approximated by $\left| \left| Y^{T}Y \right| \right|_{F}^{2}$. As entire features may or may not be removed, $q \leq p$. The full FL statistic is defined as such:

$$\begin{aligned} FL\left( J \right)= 1-\frac{\left| \left| Y^{T}Y \right| \right|_{F}^{2}}{\left| \left| X^{T}X \right| \right|_{F}^{2}}\#\left( 1 \right) \end{aligned}$$

where the statistic is the ratio between the covariances of post-filtered count matrix $Y$ and pre-filtered count matrix $X$. As post-filtering count matrix $Y$ is a subset of pre-filtering count matrix $X$, this statistic can approximate the contribution of the filtered features or counts removed from $Y$ to the covariance of the overall, pre-filtering count matrix $X$. This statistic $FL(J)$ is between 0 and 1, with values closer to 1 indicating high contribution of the filtered features or counts to the overall covariance of the pre-filtering count matrix $X$.

The pre-filtering and post-filtering count matrices will never include the negative control samples.

- - 1. **Filtering Loss Example**

With this in mind, we can create a toy example of this using the following pre-filtering $n x p$ count matrix $X$ and post-filtering $n x q$ count matrix $Y$ which is a subset of $X$. These count matrices are as follows:

$$X_{nxp}= \left[ \begin{matrix} 0 & 14 & 0 & 12 & 0 \\ 12 & 0 & 2 & 49 & 3 \\ 8 & 31 & 52 & 105 & 0 \\ 10 & 0 & 0 & 182 & 17 \\ 4 & 0 & 1 & 21 & 21 \end{matrix} \right]Y_{nxq}= \left[ \begin{matrix} 0 & 14 & 0 & 0 \\ 12 & 0 & 2 & 3 \\ 8 & 31 & 48 & 0 \\ 10 & 0 & 0 & 17 \\ 4 & 0 & 1 & 15 \end{matrix} \right]$$

In this case, we see that the pre-filtering matrix has 5 samples, $n$, and 5 features, $p$. After filtering, the post-filtering matrix retains the 5 samples, $n$, but has filtered one whole feature ($p$= 4 from $X$) and a handful of counts, resulting in 4 features, $q$. To calculate the filtering loss (FL) statistic, we must first calculate the squared Frobenius norm for each of these count matrices. For the pre-filtering matrix, this can be calculated as:

$$\begin{aligned} \left| \left| X^{T}X \right| \right|_{F}^{2}=\Sigma_{j=1}^{p}\left( x_{j}^{T}x_{j} \right)^{2}+\Sigma_{i>j}\left( x_{i}^{T}x_{j} \right)^{2}\#\left( A1.1 \right) \end{aligned}$$

$$\left| \left| X^{T}X \right| \right|_{F}^{2}\approx502064$$

and for the post filtering matrix as:

$$\begin{aligned} \left| \left| Y^{T}Y \right| \right|_{F}^{2}=\Sigma_{j=1}^{q}\left( y_{j}^{T}y_{j} \right)^{2}+\Sigma_{i>j}\left( y_{i}^{T}y_{j} \right)^{2}\#\left( A1.2 \right) \end{aligned}$$

$$\left| \left| Y^{T}Y \right| \right|_{F}^{2}\approx4313$$

We can then use these values in our FL formula to calculate the impact of filtering the one feature and handful of counts. This is calculated as such:

$$\begin{aligned} FL\left( J \right)= 1-\frac{\left| \left| Y^{T}Y \right| \right|_{F}^{2}}{\left| \left| X^{T}X \right| \right|_{F}^{2}}\#\left( 1 \right) \end{aligned}$$

$$FL\left( J \right)\approx1-\frac{4313}{502064}$$

$$FL\left( J \right)\approx0.917$$

With a filtering loss value of 0.917, we can see that the filtered feature and counts from pre-filtering count matrix $X$ has a large impact on the covariance.
